# Supplementary material for: Chromothripsis during telomere crisis is independent of NHEJ, and consistent with a replicative origin
Source: Genome Res. 2019 May;29(5):737–49. doi: 10.1101/gr.240705.118 (PMC6499312; doi:10.1101/gr.240705.118)
Supplement: Supplemental Material [file supp_gr.240705.118_Supplemental_file_1.zip › contigs/annotated_contigs/DB49/contig.2.DB49_length_928_mean_cov_9.19612068966.docx]

**DB49_length_928_mean_cov_9.19612068966**

```
AGGCCCAGAACAAGAAATTCTGTTTAGCATCTGGAGATCTATGGCCAGGCTGGATCCAAATGGATTCCAGTTTAGAGGGCTGTCCACTC
 >chr3:5048831-5049312 + E=7e-245
CTTGGCAAAGTCCTGTTTTCTTAACCCTCTCTTCCCTTTGAACAGTGATCAAGGATCCTCCCAAGTCCTTCTCTAATCCCTCGTAGCCC

CACTAACAGCCATCACAGTCAGACACCCTCCTCAGAGAGCCATTTCTCCTCCTTGGTCTTTCTCTTCTAATTTCCACTCCTGATATCTA

GCTAGCTACTTCTTCTAAACATGTCAAGGGACCTAGTTTCCAGTTCTGCTCTGGCTCTTACTCAGTGGCTTCTAGTAATTTCCAGCCCT

TCTCTGTGCCTCAGTTGCCCCATTTGTAAAATGGGGCCCCAAAAGGTTCCTTTCGGCTCTGACATTCTAGGATCCAAAGTACAGAGATT

AAGCCACAAGAATCCCTACCAGAAGGTAGGG|ATTCAGACATAAATAAGTATTGGTTCATGCTACAACATGCGTGAACCTTGAAAATAC
                                 >chr3:4496420-4496872 + E=6e-260
TATATTAAGTGAAAGAATCCAATCACAAAGGGCCACATATTGTATGAATCTATTCATATGAAATGTCCAGAATAGGCAAATCCAAAGAG

ACACAAAGTAGATTAGTGGTTGCCAGAGGCTAGGGGGAGGCAGGAATGGGGAATGATTGCTAATGGGCATAGAGTTTCTTCTTGAGATG

ATGAAGTATTCTGGAATTAGGCAGTGATAGTTGTAACACCTCATAACTATATTAAAAACCACTGAATTGTACATGTTGAAAGAGTGTAT

TGCATTCTATATGAATTCTATCTCAACTTTTTTCAAAAACCACAGGCCTTCAACCATACCATCTGTCAGAAATACACTCTTTACAGACA

CAATGTCTTATTTTCAAATCATTTGAGTGAGCCAATTAA
```
